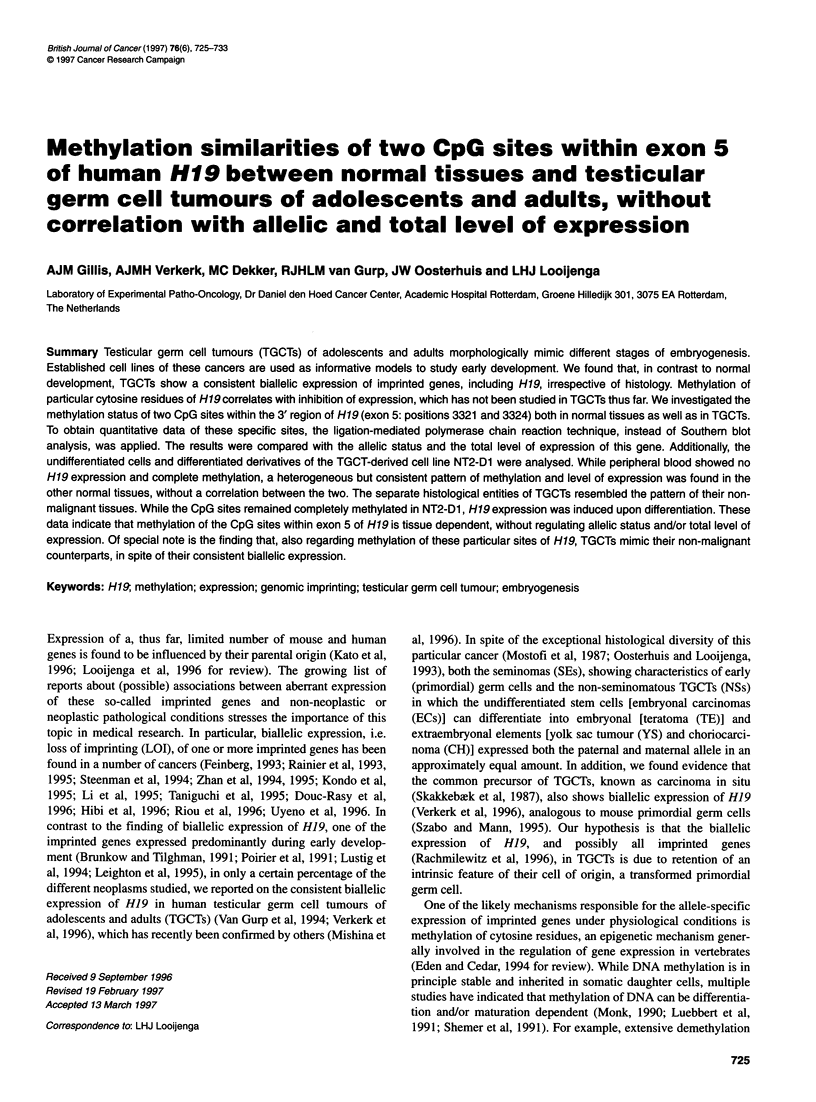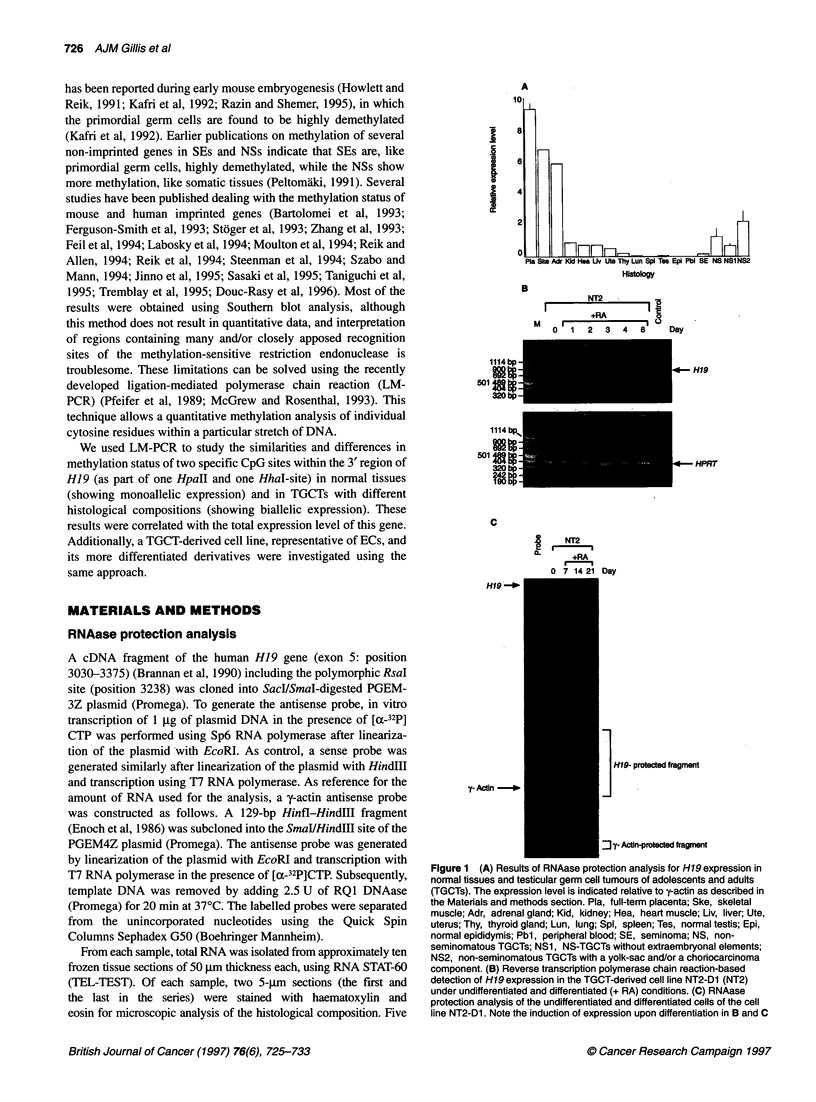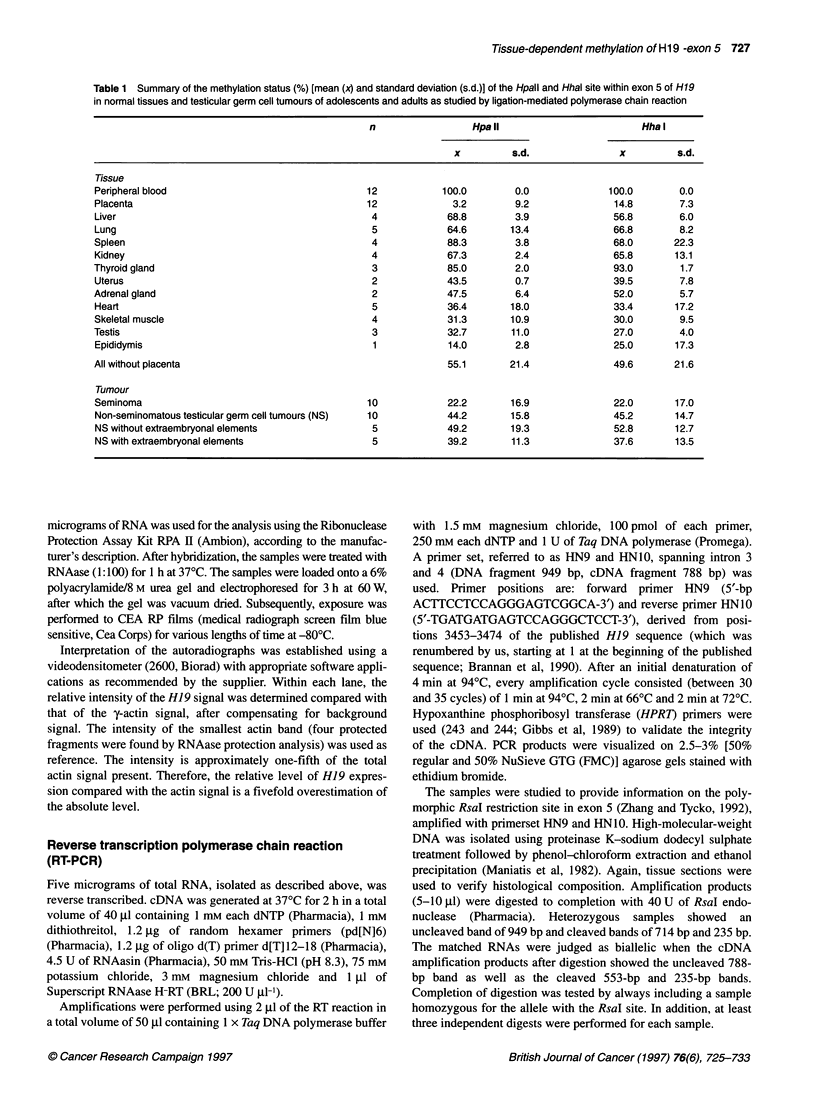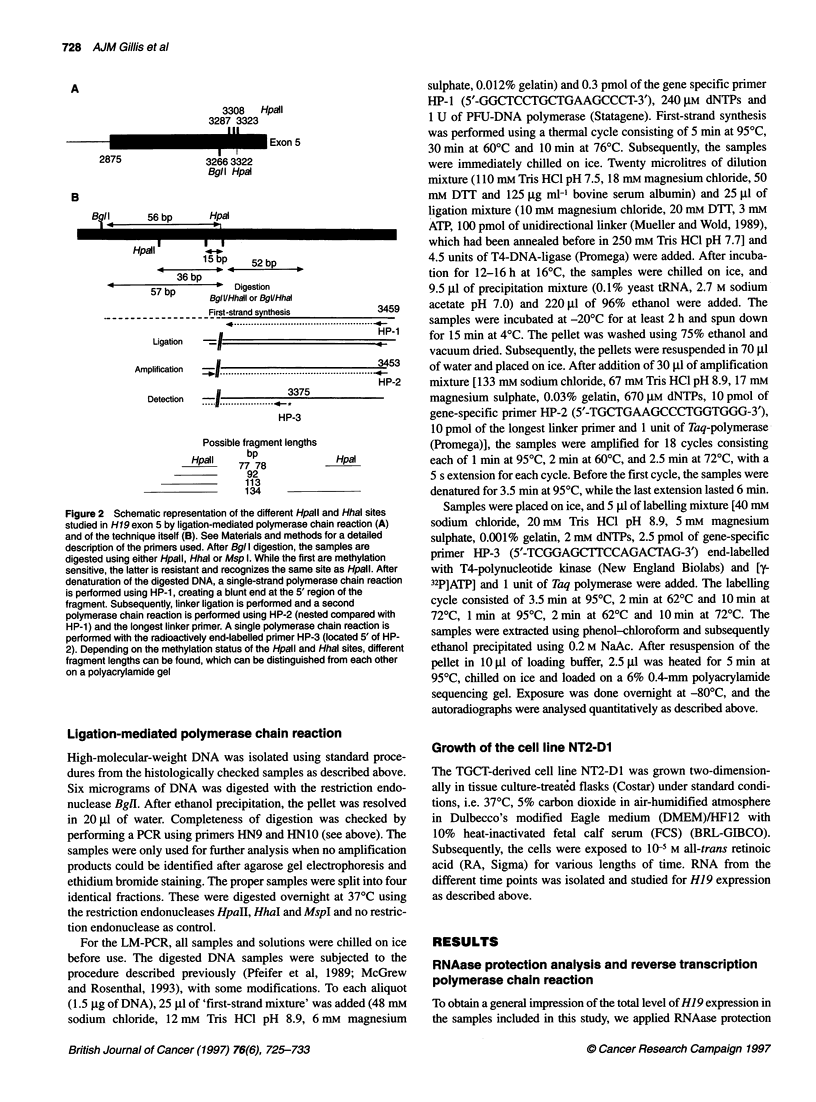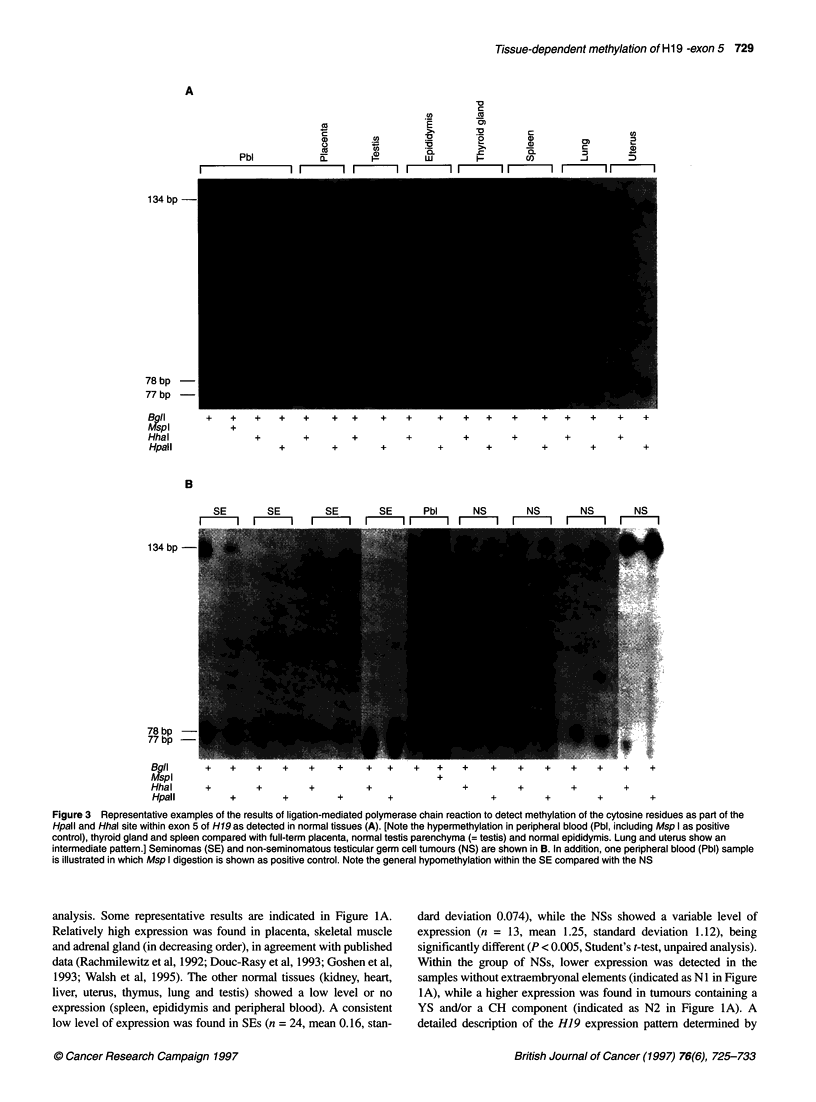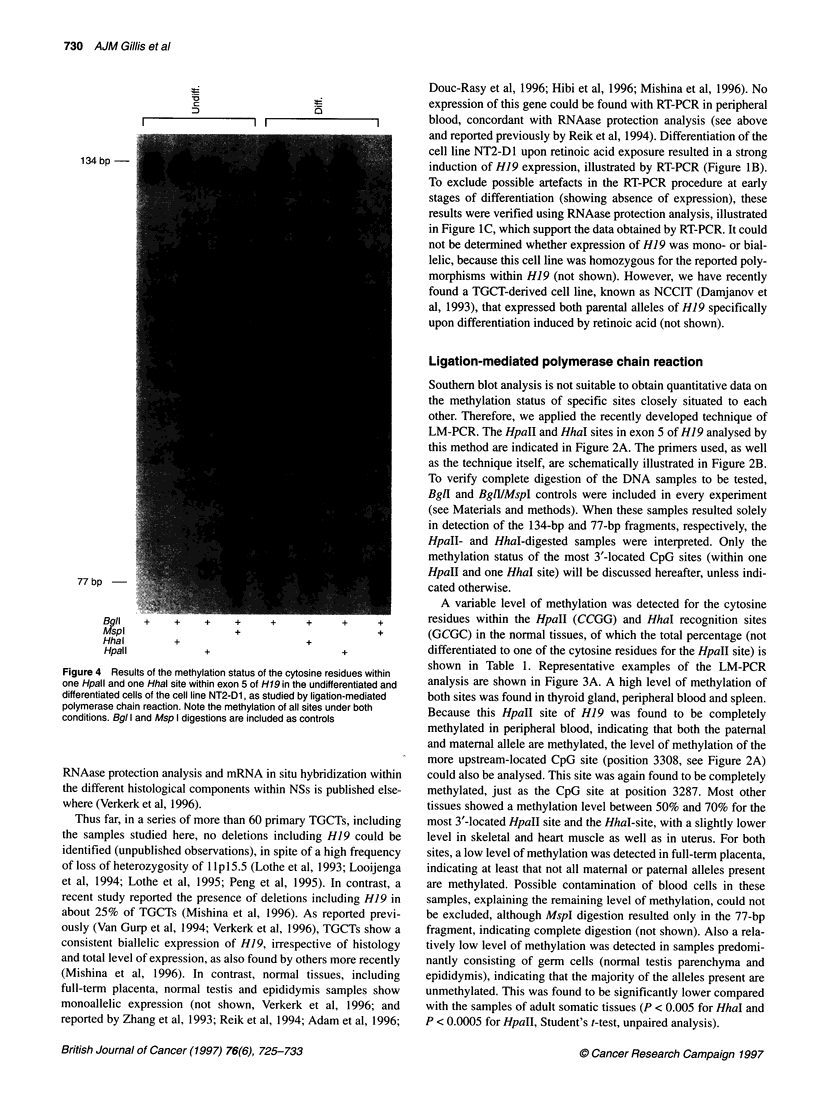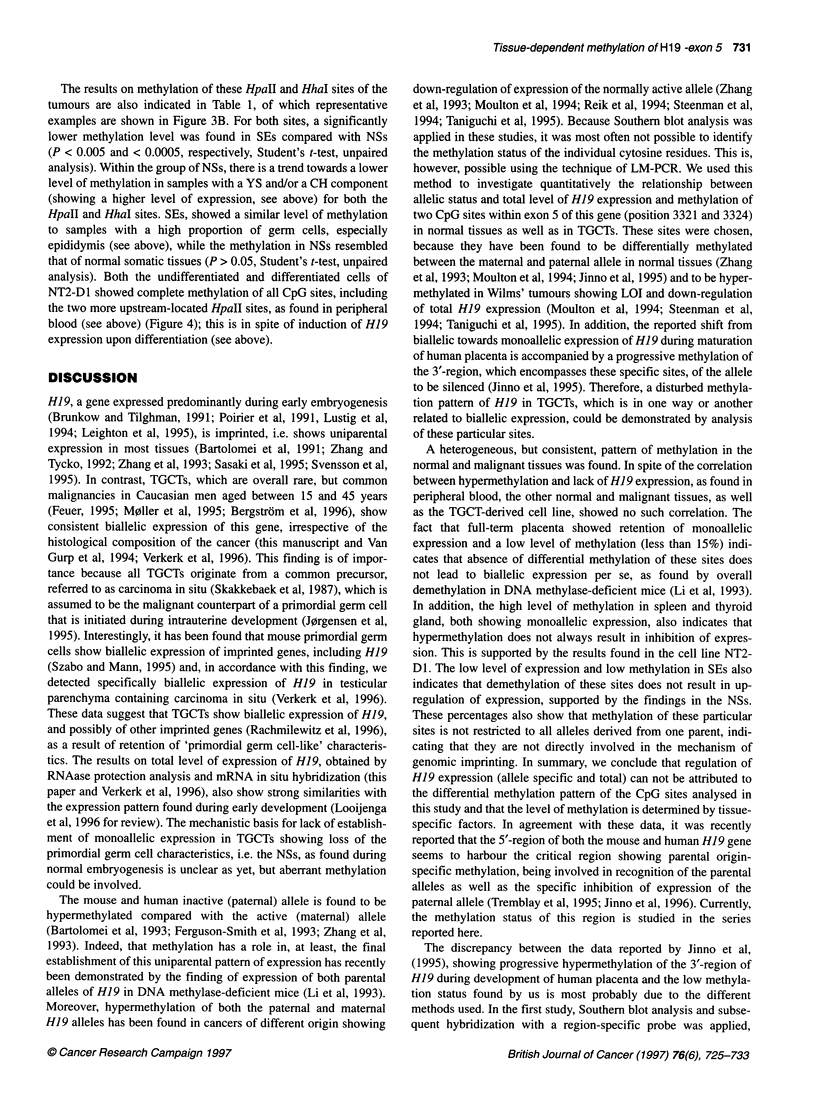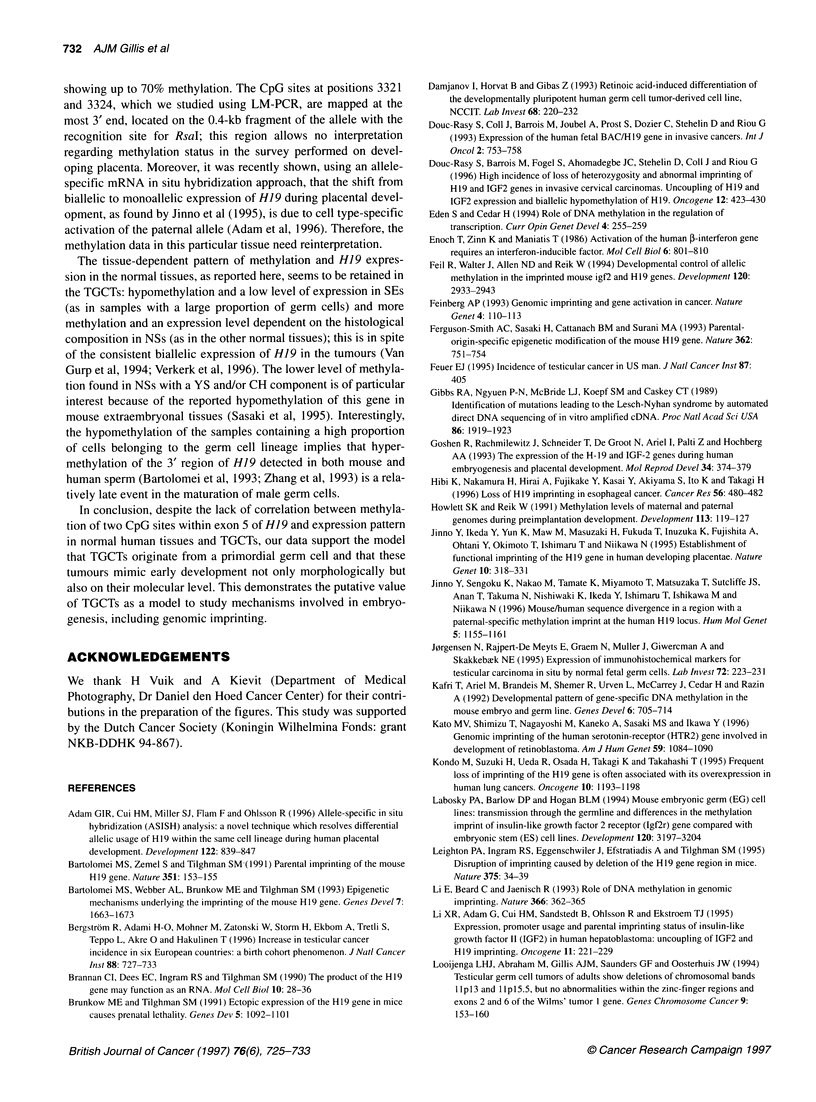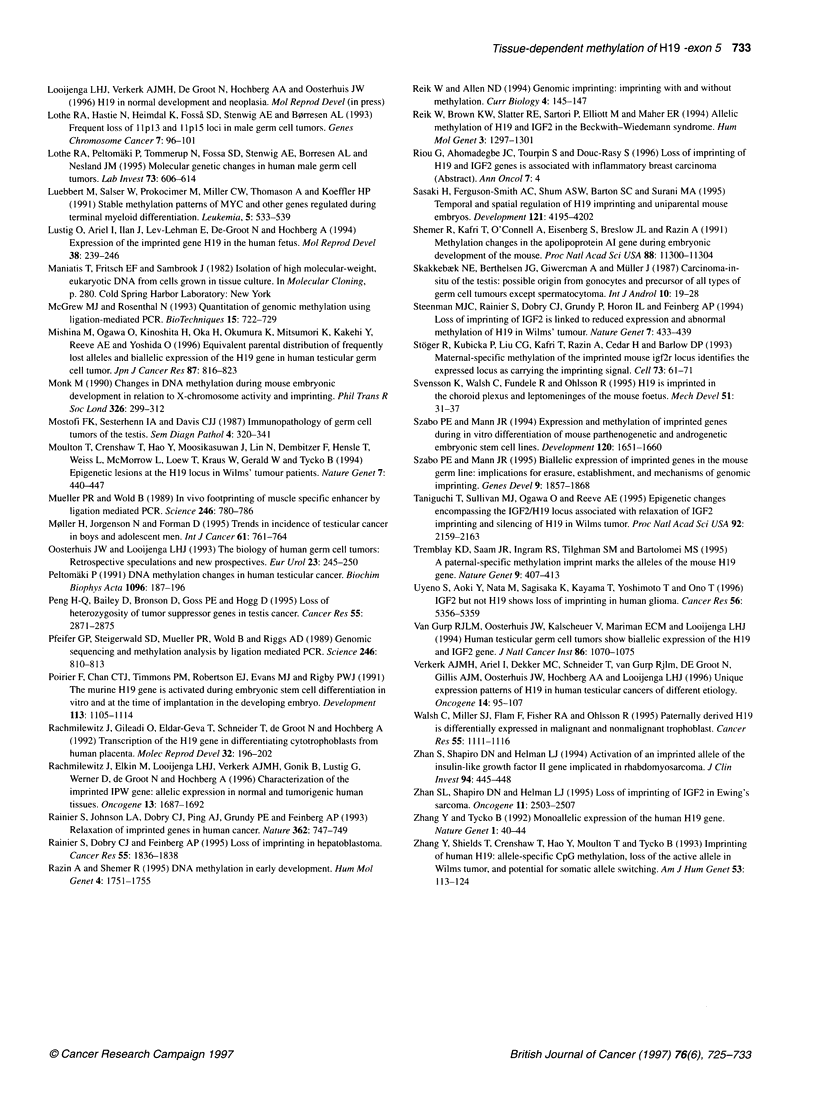# Methylation similarities of two CpG sites within exon 5 of human H19 between normal tissues and testicular germ cell tumours of adolescents and adults, without correlation with allelic and total level of expression.

**DOI:** 10.1038/bjc.1997.453

**Published:** 1997

**Authors:** A. J. Gillis, A. J. Verkerk, M. C. Dekker, R. J. van Gurp, J. W. Oosterhuis, L. H. Looijenga

**Affiliations:** Laboratory of Experimental Patho-Oncology, Dr Daniel den Hoed Cancer Center, Academic Hospital Rotterdam, The Netherlands.

## Abstract

**Images:**


British Journal of Cancer (1997) 76(6), 725–733
© 1997 Cancer Research Campaign

### Methylation similarities of two CpG sites within exon 5 of human *H19* between normal tissues and testicular germ cell tumours of adolescents and adults, without correlation with allelic and total level of expression

**AJM Gillis, AJMH Verkerk, MC Dekker, RJHLM van Gurp, JW Oosterhuis and LHJ Looijenga**

Laboratory of Experimental Patho-Oncology, Dr Daniel den Hoed Cancer Center, Academic Hospital Rotterdam, Groene Hilledijk 301, 3075 EA Rotterdam, The Netherlands

**Summary** Testicular germ cell tumours (TGCTs) of adolescents and adults morphologically mimic different stages of embryogenesis. Established cell lines of these cancers are used as informative models to study early development. We found that, in contrast to normal development, TGCTs show a consistent biallelic expression of imprinted genes, including *H19*, irrespective of histology. Methylation of particular cytosine residues of *H19* correlates with inhibition of expression, which has not been studied in TGCTs thus far. We investigated the methylation status of two CpG sites within the 3′ region of *H19* (exon 5: positions 3321 and 3324) both in normal tissues as well as in TGCTs. To obtain quantitative data of these specific sites, the ligation-mediated polymerase chain reaction technique, instead of Southern blot analysis, was applied. The results were compared with the allelic status and the total level of expression of this gene. Additionally, the undifferentiated cells and differentiated derivatives of the TGCT-derived cell line NT2-D1 were analysed. While peripheral blood showed no *H19* expression and complete methylation, a heterogeneous but consistent pattern of methylation and level of expression was found in the other normal tissues, without a correlation between the two. The separate histological entities of TGCTs resembled the pattern of their non-malignant tissues. While the CpG sites remained completely methylated in NT2-D1, *H19* expression was induced upon differentiation. These data indicate that methylation of the CpG sites within exon 5 of *H19* is tissue dependent, without regulating allelic status and/or total level of expression. Of special note is the finding that, also regarding methylation of these particular sites of *H19*, TGCTs mimic their non-malignant counterparts, in spite of their consistent biallelic expression.

**Keywords:** *H19*; methylation; expression; genomic imprinting; testicular germ cell tumour; embryogenesis

Expression of a, thus far, limited number of mouse and human genes is found to be influenced by their parental origin (Kato et al, 1996; Looijenga et al, 1996 for review). The growing list of reports about (possible) associations between aberrant expression of these so-called imprinted genes and non-neoplastic or neoplastic pathological conditions stresses the importance of this topic in medical research. In particular, biallelic expression, i.e. loss of imprinting (LOI), of one or more imprinted genes has been found in a number of cancers (Feinberg, 1993; Rainier et al, 1993, 1995; Steenman et al, 1994; Zhan et al, 1994, 1995; Kondo et al, 1995; Li et al, 1995; Taniguchi et al, 1995; Douc-Rasy et al, 1996; Hibi et al, 1996; Riou et al, 1996; Uyeno et al, 1996. In contrast to the finding of biallelic expression of *H19*, one of the imprinted genes expressed predominantly during early development (Brunkow and Tilghman, 1991; Poirier et al, 1991; Lustig et al, 1994; Leighton et al, 1995), in only a certain percentage of the different neoplasms studied, we reported on the consistent biallelic expression of *H19* in human testicular germ cell tumours of adolescents and adults (TGCTs) (Van Gurp et al, 1994; Verkerk et al, 1996), which has recently been confirmed by others (Mishina et al, 1996). In spite of the exceptional histological diversity of this particular cancer (Mostofi et al, 1987; Oosterhuis and Looijenga, 1993), both the seminomas (SEs), showing characteristics of early (primordial) germ cells and the non-seminomatous TGCTs (NSs) in which the undifferentiated stem cells [embryonal carcinomas (ECs)] can differentiate into embryonal [teratoma (TE)] and extraembryonal elements [yolk sac tumour (YS) and choriocarcinoma (CH)] expressed both the paternal and maternal allele in an approximately equal amount. In addition, we found evidence that the common precursor of TGCTs, known as carcinoma in situ (Skakkebæk et al, 1987), also shows biallelic expression of *H19* (Verkerk et al, 1996), analogous to mouse primordial germ cells (Szabo and Mann, 1995). Our hypothesis is that the biallelic expression of *H19*, and possibly all imprinted genes (Rachmilewitz et al, 1996), in TGCTs is due to retention of an intrinsic feature of their cell of origin, a transformed primordial germ cell.

*Received 9 September 1996*
*Revised 19 February 1997*
*Accepted 13 March 1997*

*Correspondence to:* LHJ Looijenga

One of the likely mechanisms responsible for the allele-specific expression of imprinted genes under physiological conditions is methylation of cytosine residues, an epigenetic mechanism generally involved in the regulation of gene expression in vertebrates (Eden and Cedar, 1994 for review). While DNA methylation is in principle stable and inherited in somatic daughter cells, multiple studies have indicated that methylation of DNA can be differentiation and/or maturation dependent (Monk, 1990; Luebbert et al, 1991; Shemer et al, 1991). For example, extensive demethylation has been reported during early mouse embryogenesis (Howlett and Reik, 1991; Kafri et al, 1992; Razin and Shemer, 1995), in which the primordial germ cells are found to be highly demethylated (Kafri et al, 1992). Earlier publications on methylation of several non-imprinted genes in SEs and NSs indicate that SEs are, like primordial germ cells, highly demethylated, while the NSs show more methylation, like somatic tissues (Peltomäki, 1991). Several studies have been published dealing with the methylation status of mouse and human imprinted genes (Bartolomei et al, 1993; Ferguson-Smith et al, 1993; Stöger et al, 1993; Zhang et al, 1993; Feil et al, 1994; Labosky et al, 1994; Moulton et al, 1994; Reik and Allen, 1994; Reik et al, 1994; Steenman et al, 1994; Szabo and Mann, 1994; Jinno et al, 1995; Sasaki et al, 1995; Taniguchi et al, 1995; Tremblay et al, 1995; Douc-Rasy et al, 1996). Most of the results were obtained using Southern blot analysis, although this method does not result in quantitative data, and interpretation of regions containing many and/or closely apposed recognition sites of the methylation-sensitive restriction endonuclease is troublesome. These limitations can be solved using the recently developed ligation-mediated polymerase chain reaction (LM-PCR) (Pfeifer et al, 1989; McGrew and Rosenthal, 1993). This technique allows a quantitative methylation analysis of individual cytosine residues within a particular stretch of DNA.

We used LM-PCR to study the similarities and differences in methylation status of two specific CpG sites within the 3′ region of *H19* (as part of one *Hpa*II and one *Hha*I-site) in normal tissues (showing monoallelic expression) and in TGCTs with different histological compositions (showing biallelic expression). These results were correlated with the total expression level of this gene. Additionally, a TGCT-derived cell line, representative of ECs, and its more differentiated derivatives were investigated using the same approach.

### MATERIALS AND METHODS

### RNAase protection analysis

A cDNA fragment of the human *H19* gene (exon 5: position 3030–3375) (Brannan et al, 1990) including the polymorphic *Rsa*I site (position 3238) was cloned into *Sac*I/*Sma*I-digested PGEM-3Z plasmid (Promega). To generate the antisense probe, in vitro transcription of 1 μg of plasmid DNA in the presence of [α-$^{32}$P] CTP was performed using Sp6 RNA polymerase after linearization of the plasmid with *Eco*RI. As control, a sense probe was generated similarly after linearization of the plasmid with *Hin*dIII and transcription using T7 RNA polymerase. As reference for the amount of RNA used for the analysis, a γ-actin antisense probe was constructed as follows. A 129-bp *Hin*fI–*Hin*dIII fragment (Enoch et al, 1986) was subcloned into the *Sma*I/*Hin*dIII site of the PGEM4Z plasmid (Promega). The antisense probe was generated by linearization of the plasmid with *Eco*RI and transcription with T7 RNA polymerase in the presence of [α-$^{32}$P]CTP. Subsequently, template DNA was removed by adding 2.5 U of RQ1 DNAase (Promega) for 20 min at 37°C. The labelled probes were separated from the unincorporated nucleotides using the Quick Spin Columns Sephadex G50 (Boehringer Mannheim).

From each sample, total RNA was isolated from approximately ten frozen tissue sections of 50 μm thickness each, using RNA STAT-60 (TEL-TEST). Of each sample, two 5-μm sections (the first and the last in the series) were stained with haematoxylin and eosin for microscopic analysis of the histological composition. Five

**Figure 1** (**A**) Results of RNAase protection analysis for *H19* expression in normal tissues and testicular germ cell tumours of adolescents and adults (TGCTs). The expression level is indicated relative to γ-actin as described in the Materials and methods section. Pla, full-term placenta; Ske, skeletal muscle; Adr, adrenal gland; Kid, kidney; Hea, heart muscle; Liv, liver; Ute, uterus; Thy, thyroid gland; Lun, lung; Spl, spleen; Tes, normal testis; Epi, normal epididymis; Pb1, peripheral blood; SE, seminoma; NS, non-seminomatous TGCTs; NS1, NS-TGCTs without extraembryonal elements; NS2, non-seminomatous TGCTs with a yolk-sac and/or a choriocarcinoma component. (**B**) Reverse transcription polymerase chain reaction-based detection of *H19* expression in the TGCT-derived cell line NT2-D1 (NT2) under undifferentiated and differentiated (+ RA) conditions. (**C**) RNAase protection analysis of the undifferentiated and differentiated cells of the cell line NT2-D1. Note the induction of expression upon differentiation in **B** and **C**

 © Cancer Research Campaign 1997

**Table 1** Summary of the methylation status (%) [mean (*x*) and standard deviation (s.d.)] of the *Hpa*II and *Hha*I site within exon 5 of *H19* in normal tissues and testicular germ cell tumours of adolescents and adults as studied by ligation-mediated polymerase chain reaction

| | *n* | *Hpa* II | | *Hha* I | |
|---|---|---|---|---|---|
| | | *x* | s.d. | *x* | s.d. |
| *Tissue* | | | | | |
| Peripheral blood | 12 | 100.0 | 0.0 | 100.0 | 0.0 |
| Placenta | 12 | 3.2 | 9.2 | 14.8 | 7.3 |
| Liver | 4 | 68.8 | 3.9 | 56.8 | 6.0 |
| Lung | 5 | 64.6 | 13.4 | 66.8 | 8.2 |
| Spleen | 4 | 88.3 | 3.8 | 68.0 | 22.3 |
| Kidney | 4 | 67.3 | 2.4 | 65.8 | 13.1 |
| Thyroid gland | 3 | 85.0 | 2.0 | 93.0 | 1.7 |
| Uterus | 2 | 43.5 | 0.7 | 39.5 | 7.8 |
| Adrenal gland | 2 | 47.5 | 6.4 | 52.0 | 5.7 |
| Heart | 5 | 36.4 | 18.0 | 33.4 | 17.2 |
| Skeletal muscle | 4 | 31.3 | 10.9 | 30.0 | 9.5 |
| Testis | 3 | 32.7 | 11.0 | 27.0 | 4.0 |
| Epididymis | 1 | 14.0 | 2.8 | 25.0 | 17.3 |
| All without placenta | | 55.1 | 21.4 | 49.6 | 21.6 |
| *Tumour* | | | | | |
| Seminoma | 10 | 22.2 | 16.9 | 22.0 | 17.0 |
| Non-seminomatous testicular germ cell tumours (NS) | 10 | 44.2 | 15.8 | 45.2 | 14.7 |
| NS without extraembryonal elements | 5 | 49.2 | 19.3 | 52.8 | 12.7 |
| NS with extraembryonal elements | 5 | 39.2 | 11.3 | 37.6 | 13.5 |

micrograms of RNA was used for the analysis using the Ribonuclease Protection Assay Kit RPA II (Ambion), according to the manufacturer's description. After hybridization, the samples were treated with RNAase (1:100) for 1 h at 37°C. The samples were loaded onto a 6% polyacrylamide/8 M urea gel and electrophoresed for 3 h at 60 W, after which the gel was vacuum dried. Subsequently, exposure was performed to CEA RP films (medical radiograph screen film blue sensitive, Cea Corps) for various lengths of time at –80°C.

Interpretation of the autoradiographs was established using a videodensitometer (2600, Biorad) with appropriate software applications as recommended by the supplier. Within each lane, the relative intensity of the *H19* signal was determined compared with that of the γ-actin signal, after compensating for background signal. The intensity of the smallest actin band (four protected fragments were found by RNAase protection analysis) was used as reference. The intensity is approximately one-fifth of the total actin signal present. Therefore, the relative level of *H19* expression compared with the actin signal is a fivefold overestimation of the absolute level.

### Reverse transcription polymerase chain reaction (RT-PCR)

Five micrograms of total RNA, isolated as described above, was reverse transcribed. cDNA was generated at 37°C for 2 h in a total volume of 40 μl containing 1 mM each dNTP (Pharmacia), 1 mM dithiothreitol, 1.2 μg of random hexamer primers (pd[N]6) (Pharmacia), 1.2 μg of oligo d(T) primer d[T]12–18 (Pharmacia), 4.5 U of RNAasin (Pharmacia), 50 mM Tris-HCl (pH 8.3), 75 mM potassium chloride, 3 mM magnesium chloride and 1 μl of Superscript RNAase H-RT (BRL; 200 U $\mu l^{-1}$).

Amplifications were performed using 2 μl of the RT reaction in a total volume of 50 μl containing 1 × *Taq* DNA polymerase buffer with 1.5 mM magnesium chloride, 100 pmol of each primer, 250 mM each dNTP and 1 U of *Taq* DNA polymerase (Promega). A primer set, referred to as HN9 and HN10, spanning intron 3 and 4 (DNA fragment 949 bp, cDNA fragment 788 bp) was used. Primer positions are: forward primer HN9 (5′-bp ACTTCCTCCAGGGAGTCGGCA-3′) and reverse primer HN10 (5′-TGATGATGAGTCCAGGGCTCCT-3′), derived from positions 3453–3474 of the published *H19* sequence (which was renumbered by us, starting at 1 at the beginning of the published sequence; Brannan et al, 1990). After an initial denaturation of 4 min at 94°C, every amplification cycle consisted (between 30 and 35 cycles) of 1 min at 94°C, 2 min at 66°C and 2 min at 72°C. Hypoxanthine phosphoribosyl transferase (*HPRT*) primers were used (243 and 244; Gibbs et al, 1989) to validate the integrity of the cDNA. PCR products were visualized on 2.5–3% [50% regular and 50% NuSieve GTG (FMC)] agarose gels stained with ethidium bromide.

The samples were studied to provide information on the polymorphic *Rsa*I restriction site in exon 5 (Zhang and Tycko, 1992), amplified with primerset HN9 and HN10. High-molecular-weight DNA was isolated using proteinase K–sodium dodecyl sulphate treatment followed by phenol–chloroform extraction and ethanol precipitation (Maniatis et al, 1982). Again, tissue sections were used to verify histological composition. Amplification products (5–10 μl) were digested to completion with 40 U of *Rsa*I endonuclease (Pharmacia). Heterozygous samples showed an uncleaved band of 949 bp and cleaved bands of 714 bp and 235 bp. The matched RNAs were judged as biallelic when the cDNA amplification products after digestion showed the uncleaved 788-bp band as well as the cleaved 553-bp and 235-bp bands. Completion of digestion was tested by always including a sample homozygous for the allele with the *Rsa*I site. In addition, at least three independent digests were performed for each sample.

© Cancer Research Campaign 1997

**Figure 2** Schematic representation of the different *Hpa*II and *Hha*I sites studied in *H19* exon 5 by ligation-mediated polymerase chain reaction (**A**) and of the technique itself (**B**). See Materials and methods for a detailed description of the primers used. After *Bgl* I digestion, the samples are digested using either *Hpa*II, *Hha*I or *Msp* I. While the first are methylation sensitive, the latter is resistant and recognizes the same site as *Hpa*II. After denaturation of the digested DNA, a single-strand polymerase chain reaction is performed using HP-1, creating a blunt end at the 5′ region of the fragment. Subsequently, linker ligation is performed and a second polymerase chain reaction is performed using HP-2 (nested compared with HP-1) and the longest linker primer. A single polymerase chain reaction is performed with the radioactively end-labelled primer HP-3 (located 5′ of HP-2). Depending on the methylation status of the *Hpa*II and *Hha*I sites, different fragment lengths can be found, which can be distinguished from each other on a polyacrylamide gel

### Ligation-mediated polymerase chain reaction

High-molecular-weight DNA was isolated using standard procedures from the histologically checked samples as described above. Six micrograms of DNA was digested with the restriction endonuclease *Bgl*I. After ethanol precipitation, the pellet was resolved in 20 µl of water. Completeness of digestion was checked by performing a PCR using primers HN9 and HN10 (see above). The samples were only used for further analysis when no amplification products could be identified after agarose gel electrophoresis and ethidium bromide staining. The proper samples were split into four identical fractions. These were digested overnight at 37°C using the restriction endonucleases *Hpa*II, *Hha*I and *Msp*I and no restriction endonuclease as control.

For the LM-PCR, all samples and solutions were chilled on ice before use. The digested DNA samples were subjected to the procedure described previously (Pfeifer et al, 1989; McGrew and Rosenthal, 1993), with some modifications. To each aliquot (1.5 µg of DNA), 25 µl of 'first-strand mixture' was added (48 mM sodium chloride, 12 mM Tris HCl pH 8.9, 6 mM magnesium sulphate, 0.012% gelatin) and 0.3 pmol of the gene specific primer HP-1 (5′-GGCTCCTGCTGAAGCCCT-3′), 240 µM dNTPs and 1 U of PFU-DNA polymerase (Statagene). First-strand synthesis was performed using a thermal cycle consisting of 5 min at 95°C, 30 min at 60°C and 10 min at 76°C. Subsequently, the samples were immediately chilled on ice. Twenty microlitres of dilution mixture (110 mM Tris HCl pH 7.5, 18 mM magnesium chloride, 50 mM DTT and 125 µg $ml^{-1}$ bovine serum albumin) and 25 µl of ligation mixture (10 mM magnesium chloride, 20 mM DTT, 3 mM ATP, 100 pmol of unidirectional linker (Mueller and Wold, 1989), which had been annealed before in 250 mM Tris HCl pH 7.7] and 4.5 units of T4-DNA-ligase (Promega) were added. After incubation for 12–16 h at 16°C, the samples were chilled on ice, and 9.5 µl of precipitation mixture (0.1% yeast tRNA, 2.7 M sodium acetate pH 7.0) and 220 µl of 96% ethanol were added. The samples were incubated at –20°C for at least 2 h and spun down for 15 min at 4°C. The pellet was washed using 75% ethanol and vacuum dried. Subsequently, the pellets were resuspended in 70 µl of water and placed on ice. After addition of 30 µl of amplification mixture [133 mM sodium chloride, 67 mM Tris HCl pH 8.9, 17 mM magnesium sulphate, 0.03% gelatin, 670 µM dNTPs, 10 pmol of gene-specific primer HP-2 (5′-TGCTGAAGCCCTGGTGGG-3′), 10 pmol of the longest linker primer and 1 unit of *Taq*-polymerase (Promega)], the samples were amplified for 18 cycles consisting each of 1 min at 95°C, 2 min at 60°C, and 2.5 min at 72°C, with a 5 s extension for each cycle. Before the first cycle, the samples were denatured for 3.5 min at 95°C, while the last extension lasted 6 min.

Samples were placed on ice, and 5 µl of labelling mixture [40 mM sodium chloride, 20 mM Tris HCl pH 8.9, 5 mM magnesium sulphate, 0.001% gelatin, 2 mM dNTPs, 2.5 pmol of gene-specific primer HP-3 (5′-TCGGAGCTTCCAGACTAG-3′) end-labelled with T4-polynucleotide kinase (New England Biolabs) and [γ-$^{32}$P]ATP] and 1 unit of *Taq* polymerase were added. The labelling cycle consisted of 3.5 min at 95°C, 2 min at 62°C and 10 min at 72°C, 1 min at 95°C, 2 min at 62°C and 10 min at 72°C. The samples were extracted using phenol–chloroform and subsequently ethanol precipitated using 0.2 M NaAc. After resuspension of the pellet in 10 µl of loading buffer, 2.5 µl was heated for 5 min at 95°C, chilled on ice and loaded on a 6% 0.4-mm polyacrylamide sequencing gel. Exposure was done overnight at –80°C, and the autoradiographs were analysed quantitatively as described above.

### Growth of the cell line NT2-D1

The TGCT-derived cell line NT2-D1 was grown two-dimensionally in tissue culture-treated flasks (Costar) under standard conditions, i.e. 37°C, 5% carbon dioxide in air-humidified atmosphere in Dulbecco's modified Eagle medium (DMEM)/HF12 with 10% heat-inactivated fetal calf serum (FCS) (BRL-GIBCO). Subsequently, the cells were exposed to $10^{-5}$ M all-*trans* retinoic acid (RA, Sigma) for various lengths of time. RNA from the different time points was isolated and studied for *H19* expression as described above.

### RESULTS

### RNAase protection analysis and reverse transcription polymerase chain reaction

To obtain a general impression of the total level of *H19* expression in the samples included in this study, we applied RNAase protection

 © Cancer Research Campaign 1997

**Figure 3** Representative examples of the results of ligation-mediated polymerase chain reaction to detect methylation of the cytosine residues as part of the *Hpa*II and *Hha*I site within exon 5 of *H19* as detected in normal tissues (**A**). [Note the hypermethylation in peripheral blood (Pbl, including *Msp* I as positive control), thyroid gland and spleen compared with full-term placenta, normal testis parenchyma (= testis) and normal epididymis. Lung and uterus show an intermediate pattern.] Seminomas (SE) and non-seminomatous testicular germ cell tumours (NS) are shown in **B**. In addition, one peripheral blood (Pbl) sample is illustrated in which *Msp* I digestion is shown as positive control. Note the general hypomethylation within the SE compared with the NS

analysis. Some representative results are indicated in Figure 1A. Relatively high expression was found in placenta, skeletal muscle and adrenal gland (in decreasing order), in agreement with published data (Rachmilewitz et al, 1992; Douc-Rasy et al, 1993; Goshen et al, 1993; Walsh et al, 1995). The other normal tissues (kidney, heart, liver, uterus, thymus, lung and testis) showed a low level or no expression (spleen, epididymis and peripheral blood). A consistent low level of expression was found in SEs ($n$ = 24, mean 0.16, standard deviation 0.074), while the NSs showed a variable level of expression ($n$ = 13, mean 1.25, standard deviation 1.12), being significantly different ($P < 0.005$, Student's $t$-test, unpaired analysis). Within the group of NSs, lower expression was detected in the samples without extraembryonal elements (indicated as N1 in Figure 1A), while a higher expression was found in tumours containing a YS and/or a CH component (indicated as N2 in Figure 1A). A detailed description of the *H19* expression pattern determined by

© Cancer Research Campaign 1997

**Figure 4** Results of the methylation status of the cytosine residues within one *Hpa*II and one *Hha*I site within exon 5 of *H19* in the undifferentiated and differentiated cells of the cell line NT2-D1, as studied by ligation-mediated polymerase chain reaction. Note the methylation of all sites under both conditions. *Bgl* I and *Msp* I digestions are included as controls

RNAase protection analysis and mRNA in situ hybridization within the different histological components within NSs is published elsewhere (Verkerk et al, 1996).

Thus far, in a series of more than 60 primary TGCTs, including the samples studied here, no deletions including *H19* could be identified (unpublished observations), in spite of a high frequency of loss of heterozygosity of 11p15.5 (Lothe et al, 1993; Looijenga et al, 1994; Lothe et al, 1995; Peng et al, 1995). In contrast, a recent study reported the presence of deletions including *H19* in about 25% of TGCTs (Mishina et al, 1996). As reported previously (Van Gurp et al, 1994; Verkerk et al, 1996), TGCTs show a consistent biallelic expression of *H19*, irrespective of histology and total level of expression, as also found by others more recently (Mishina et al, 1996). In contrast, normal tissues, including full-term placenta, normal testis and epididymis samples show monoallelic expression (not shown, Verkerk et al, 1996; and reported by Zhang et al, 1993; Reik et al, 1994; Adam et al, 1996; Douc-Rasy et al, 1996; Hibi et al, 1996; Mishina et al, 1996). No expression of this gene could be found with RT-PCR in peripheral blood, concordant with RNAase protection analysis (see above and reported previously by Reik et al, 1994). Differentiation of the cell line NT2-D1 upon retinoic acid exposure resulted in a strong induction of *H19* expression, illustrated by RT-PCR (Figure 1B). To exclude possible artefacts in the RT-PCR procedure at early stages of differentiation (showing absence of expression), these results were verified using RNAase protection analysis, illustrated in Figure 1C, which support the data obtained by RT-PCR. It could not be determined whether expression of *H19* was mono- or biallelic, because this cell line was homozygous for the reported polymorphisms within *H19* (not shown). However, we have recently found a TGCT-derived cell line, known as NCCIT (Damjanov et al, 1993), that expressed both parental alleles of *H19* specifically upon differentiation induced by retinoic acid (not shown).

### Ligation-mediated polymerase chain reaction

Southern blot analysis is not suitable to obtain quantitative data on the methylation status of specific sites closely situated to each other. Therefore, we applied the recently developed technique of LM-PCR. The *Hpa*II and *Hha*I sites in exon 5 of *H19* analysed by this method are indicated in Figure 2A. The primers used, as well as the technique itself, are schematically illustrated in Figure 2B. To verify complete digestion of the DNA samples to be tested, *Bgl*I and *Bgl*I/*Msp*I controls were included in every experiment (see Materials and methods). When these samples resulted solely in detection of the 134-bp and 77-bp fragments, respectively, the *Hpa*II- and *Hha*I-digested samples were interpreted. Only the methylation status of the most 3′-located CpG sites (within one *Hpa*II and one *Hha*I site) will be discussed hereafter, unless indicated otherwise.

A variable level of methylation was detected for the cytosine residues within the *Hpa*II (*C*CGG) and *Hha*I recognition sites (G*C*GC) in the normal tissues, of which the total percentage (not differentiated to one of the cytosine residues for the *Hpa*II site) is shown in Table 1. Representative examples of the LM-PCR analysis are shown in Figure 3A. A high level of methylation of both sites was found in thyroid gland, peripheral blood and spleen. Because this *Hpa*II site of *H19* was found to be completely methylated in peripheral blood, indicating that both the paternal and maternal allele are methylated, the level of methylation of the more upstream-located CpG site (position 3308, see Figure 2A) could also be analysed. This site was again found to be completely methylated, just as the CpG site at position 3287. Most other tissues showed a methylation level between 50% and 70% for the most 3′-located *Hpa*II site and the *Hha*I-site, with a slightly lower level in skeletal and heart muscle as well as in uterus. For both sites, a low level of methylation was detected in full-term placenta, indicating at least that not all maternal or paternal alleles present are methylated. Possible contamination of blood cells in these samples, explaining the remaining level of methylation, could not be excluded, although *Msp*I digestion resulted only in the 77-bp fragment, indicating complete digestion (not shown). Also a relatively low level of methylation was detected in samples predominantly consisting of germ cells (normal testis parenchyma and epididymis), indicating that the majority of the alleles present are unmethylated. This was found to be significantly lower compared with the samples of adult somatic tissues ($P < 0.005$ for *Hha*I and $P < 0.0005$ for *Hpa*II, Student's *t*-test, unpaired analysis).

 *© Cancer Research Campaign 1997*

The results on methylation of these *Hpa*II and *Hha*I sites of the tumours are also indicated in Table 1, of which representative examples are shown in Figure 3B. For both sites, a significantly lower methylation level was found in SEs compared with NSs ($P < 0.005$ and $< 0.0005$, respectively, Student's *t*-test, unpaired analysis). Within the group of NSs, there is a trend towards a lower level of methylation in samples with a YS and/or a CH component (showing a higher level of expression, see above) for both the *Hpa*II and *Hha*I sites. SEs, showed a similar level of methylation to samples with a high proportion of germ cells, especially epididymis (see above), while the methylation in NSs resembled that of normal somatic tissues ($P > 0.05$, Student's *t*-test, unpaired analysis). Both the undifferentiated and differentiated cells of NT2-D1 showed complete methylation of all CpG sites, including the two more upstream-located *Hpa*II sites, as found in peripheral blood (see above) (Figure 4); this is in spite of induction of *H19* expression upon differentiation (see above).

### DISCUSSION

*H19*, a gene expressed predominantly during early embryogenesis (Brunkow and Tilghman, 1991; Poirier et al, 1991, Lustig et al, 1994; Leighton et al, 1995), is imprinted, i.e. shows uniparental expression in most tissues (Bartolomei et al, 1991; Zhang and Tycko, 1992; Zhang et al, 1993; Sasaki et al, 1995; Svensson et al, 1995). In contrast, TGCTs, which are overall rare, but common malignancies in Caucasian men aged between 15 and 45 years (Feuer, 1995; Møller et al, 1995; Bergström et al, 1996), show consistent biallelic expression of this gene, irrespective of the histological composition of the cancer (this manuscript and Van Gurp et al, 1994; Verkerk et al, 1996). This finding is of importance because all TGCTs originate from a common precursor, referred to as carcinoma in situ (Skakkebaek et al, 1987), which is assumed to be the malignant counterpart of a primordial germ cell that is initiated during intrauterine development (Jørgensen et al, 1995). Interestingly, it has been found that mouse primordial germ cells show biallelic expression of imprinted genes, including *H19* (Szabo and Mann, 1995) and, in accordance with this finding, we detected specifically biallelic expression of *H19* in testicular parenchyma containing carcinoma in situ (Verkerk et al, 1996). These data suggest that TGCTs show biallelic expression of *H19*, and possibly of other imprinted genes (Rachmilewitz et al, 1996), as a result of retention of 'primordial germ cell-like' characteristics. The results on total level of expression of *H19*, obtained by RNAase protection analysis and mRNA in situ hybridization (this paper and Verkerk et al, 1996), also show strong similarities with the expression pattern found during early development (Looijenga et al, 1996 for review). The mechanistic basis for lack of establishment of monoallelic expression in TGCTs showing loss of the primordial germ cell characteristics, i.e. the NSs, as found during normal embryogenesis is unclear as yet, but aberrant methylation could be involved.

The mouse and human inactive (paternal) allele is found to be hypermethylated compared with the active (maternal) allele (Bartolomei et al, 1993; Ferguson-Smith et al, 1993; Zhang et al, 1993). Indeed, that methylation has a role in, at least, the final establishment of this uniparental pattern of expression has recently been demonstrated by the finding of expression of both parental alleles of *H19* in DNA methylase-deficient mice (Li et al, 1993). Moreover, hypermethylation of both the paternal and maternal *H19* alleles has been found in cancers of different origin showing down-regulation of expression of the normally active allele (Zhang et al, 1993; Moulton et al, 1994; Reik et al, 1994; Steenman et al, 1994; Taniguchi et al, 1995). Because Southern blot analysis was applied in these studies, it was most often not possible to identify the methylation status of the individual cytosine residues. This is, however, possible using the technique of LM-PCR. We used this method to investigate quantitatively the relationship between allelic status and total level of *H19* expression and methylation of two CpG sites within exon 5 of this gene (position 3321 and 3324) in normal tissues as well as in TGCTs. These sites were chosen, because they have been found to be differentially methylated between the maternal and paternal allele in normal tissues (Zhang et al, 1993; Moulton et al, 1994; Jinno et al, 1995) and to be hypermethylated in Wilms' tumours showing LOI and down-regulation of total *H19* expression (Moulton et al, 1994; Steenman et al, 1994; Taniguchi et al, 1995). In addition, the reported shift from biallelic towards monoallelic expression of *H19* during maturation of human placenta is accompanied by a progressive methylation of the 3′-region, which encompasses these specific sites, of the allele to be silenced (Jinno et al, 1995). Therefore, a disturbed methylation pattern of *H19* in TGCTs, which is in one way or another related to biallelic expression, could be demonstrated by analysis of these particular sites.

A heterogeneous, but consistent, pattern of methylation in the normal and malignant tissues was found. In spite of the correlation between hypermethylation and lack of *H19* expression, as found in peripheral blood, the other normal and malignant tissues, as well as the TGCT-derived cell line, showed no such correlation. The fact that full-term placenta showed retention of monoallelic expression and a low level of methylation (less than 15%) indicates that absence of differential methylation of these sites does not lead to biallelic expression per se, as found by overall demethylation in DNA methylase-deficient mice (Li et al, 1993). In addition, the high level of methylation in spleen and thyroid gland, both showing monoallelic expression, also indicates that hypermethylation does not always result in inhibition of expression. This is supported by the results found in the cell line NT2-D1. The low level of expression and low methylation in SEs also indicates that demethylation of these sites does not result in up-regulation of expression, supported by the findings in the NSs. These percentages also show that methylation of these particular sites is not restricted to all alleles derived from one parent, indicating that they are not directly involved in the mechanism of genomic imprinting. In summary, we conclude that regulation of *H19* expression (allele specific and total) can not be attributed to the differential methylation pattern of the CpG sites analysed in this study and that the level of methylation is determined by tissue-specific factors. In agreement with these data, it was recently reported that the 5′-region of both the mouse and human *H19* gene seems to harbour the critical region showing parental origin-specific methylation, being involved in recognition of the parental alleles as well as the specific inhibition of expression of the paternal allele (Tremblay et al, 1995; Jinno et al, 1996). Currently, the methylation status of this region is studied in the series reported here.

The discrepancy between the data reported by Jinno et al, (1995), showing progressive hypermethylation of the 3′-region of *H19* during development of human placenta and the low methylation status found by us is most probably due to the different methods used. In the first study, Southern blot analysis and subsequent hybridization with a region-specific probe was applied,

© Cancer Research Campaign 1997

showing up to 70% methylation. The CpG sites at positions 3321 and 3324, which we studied using LM-PCR, are mapped at the most 3′ end, located on the 0.4-kb fragment of the allele with the recognition site for *Rsa*I; this region allows no interpretation regarding methylation status in the survey performed on developing placenta. Moreover, it was recently shown, using an allele-specific mRNA in situ hybridization approach, that the shift from biallelic to monoallelic expression of *H19* during placental development, as found by Jinno et al (1995), is due to cell type-specific activation of the paternal allele (Adam et al, 1996). Therefore, the methylation data in this particular tissue need reinterpretation.

The tissue-dependent pattern of methylation and *H19* expression in the normal tissues, as reported here, seems to be retained in the TGCTs: hypomethylation and a low level of expression in SEs (as in samples with a large proportion of germ cells) and more methylation and an expression level dependent on the histological composition in NSs (as in the other normal tissues); this is in spite of the consistent biallelic expression of *H19* in the tumours (Van Gurp et al, 1994; Verkerk et al, 1996). The lower level of methylation found in NSs with a YS and/or CH component is of particular interest because of the reported hypomethylation of this gene in mouse extraembryonal tissues (Sasaki et al, 1995). Interestingly, the hypomethylation of the samples containing a high proportion of cells belonging to the germ cell lineage implies that hypermethylation of the 3′ region of *H19* detected in both mouse and human sperm (Bartolomei et al, 1993; Zhang et al, 1993) is a relatively late event in the maturation of male germ cells.

In conclusion, despite the lack of correlation between methylation of two CpG sites within exon 5 of *H19* and expression pattern in normal human tissues and TGCTs, our data support the model that TGCTs originate from a primordial germ cell and that these tumours mimic early development not only morphologically but also on their molecular level. This demonstrates the putative value of TGCTs as a model to study mechanisms involved in embryogenesis, including genomic imprinting.

### ACKNOWLEDGEMENTS

We thank H Vuik and A Kievit (Department of Medical Photography, Dr Daniel den Hoed Cancer Center) for their contributions in the preparation of the figures. This study was supported by the Dutch Cancer Society (Koningin Wilhelmina Fonds: grant NKB-DDHK 94-867).

### REFERENCES

Adam GIR, Cui HM, Miller SJ, Flam F and Ohlsson R (1996) Allele-specific in situ hybridization (ASISH) analysis: a novel technique which resolves differential allelic usage of H19 within the same cell lineage during human placental development. *Development* **122**: 839–847

Bartolomei MS, Zemel S and Tilghman SM (1991) Parental imprinting of the mouse H19 gene. *Nature* **351**: 153–155

Bartolomei MS, Webber AL, Brunkow ME and Tilghman SM (1993) Epigenetic mechanisms underlying the imprinting of the mouse H19 gene. *Genes Devel* **7**: 1663–1673

Bergström R, Adami H-O, Mohner M, Zatonski W, Storm H, Ekbom A, Tretli S, Teppo L, Akre O and Hakulinen T (1996) Increase in testicular cancer incidence in six European countries: a birth cohort phenomenon. *J Natl Cancer Inst* **88**: 727–733

Brannan CI, Dees EC, Ingram RS and Tilghman SM (1990) The product of the H19 gene may function as an RNA. *Mol Cell Biol* **10**: 28–36

Brunkow ME and Tilghman SM (1991) Ectopic expression of the H19 gene in mice causes prenatal lethality. *Genes Dev* **5**: 1092–1101

Damjanov I, Horvat B and Gibas Z (1993) Retinoic acid-induced differentiation of the developmentally pluripotent human germ cell tumor-derived cell line, NCCIT. *Lab Invest* **68**: 220–232

Douc-Rasy S, Coll J, Barrois M, Joubel A, Prost S, Dozier C, Stehelin D and Riou G (1993) Expression of the human fetal BAC/H19 gene in invasive cancers. *Int J Oncol* **2**: 753–758

Douc-Rasy S, Barrois M, Fogel S, Ahomadegbe JC, Stehelin D, Coll J and Riou G (1996) High incidence of loss of heterozygosity and abnormal imprinting of H19 and IGF2 genes in invasive cervical carcinomas. Uncoupling of H19 and IGF2 expression and biallelic hypomethylation of H19. *Oncogene* **12**: 423–430

Eden S and Cedar H (1994) Role of DNA methylation in the regulation of transcription. *Curr Opin Genet Devel* **4**: 255–259

Enoch T, Zinn K and Maniatis T (1986) Activation of the human β-interferon gene requires an interferon-inducible factor. *Mol Cell Biol* **6**: 801–810

Feil R, Walter J, Allen ND and Reik W (1994) Developmental control of allelic methylation in the imprinted mouse igf2 and H19 genes. *Development* **120**: 2933–2943

Feinberg AP (1993) Genomic imprinting and gene activation in cancer. *Nature Genet* **4**: 110–113

Ferguson-Smith AC, Sasaki H, Cattanach BM and Surani MA (1993) Parental-origin-specific epigenetic modification of the mouse H19 gene. *Nature* **362**: 751–754

Feuer EJ (1995) Incidence of testicular cancer in US man. *J Natl Cancer Inst* **87**: 405

Gibbs RA, Ngyuen P-N, McBride LJ, Koepf SM and Caskey CT (1989) Identification of mutations leading to the Lesch-Nyhan syndrome by automated direct DNA sequencing of in vitro amplified cDNA. *Proc Natl Acad Sci USA* **86**: 1919–1923

Goshen R, Rachmilewitz J, Schneider T, De Groot N, Ariel I, Palti Z and Hochberg AA (1993) The expression of the H-19 and IGF-2 genes during human embryogenesis and placental development. *Mol Reprod Devel* **34**: 374–379

Hibi K, Nakamura H, Hirai A, Fujikake Y, Kasai Y, Akiyama S, Ito K and Takagi H (1996) Loss of H19 imprinting in esophageal cancer. *Cancer Res* **56**: 480–482

Howlett SK and Reik W (1991) Methylation levels of maternal and paternal genomes during preimplantation development. *Development* **113**: 119–127

Jinno Y, Ikeda Y, Yun K, Maw M, Masuzaki H, Fukuda T, Inuzuka K, Fujishita A, Ohtani Y, Okimoto T, Ishimaru T and Niikawa N (1995) Establishment of functional imprinting of the H19 gene in human developing placentae. *Nature Genet* **10**: 318–331

Jinno Y, Sengoku K, Nakao M, Tamate K, Miyamoto T, Matsuzaka T, Sutcliffe JS, Anan T, Takuma N, Nishiwaki K, Ikeda Y, Ishimaru T, Ishikawa M and Niikawa N (1996) Mouse/human sequence divergence in a region with a paternal-specific methylation imprint at the human H19 locus. *Hum Mol Genet* **5**: 1155–1161

Jørgensen N, Rajpert-De Meyts E, Graem N, Muller J, Giwercman A and Skakkebæk NE (1995) Expression of immunohistochemical markers for testicular carcinoma in situ by normal fetal germ cells. *Lab Invest* **72**: 223–231

Kafri T, Ariel M, Brandeis M, Shemer R, Urven L, McCarrey J, Cedar H and Razin A (1992) Developmental pattern of gene-specific DNA methylation in the mouse embryo and germ line. *Genes Devel* **6**: 705–714

Kato MV, Shimizu T, Nagayoshi M, Kaneko A, Sasaki MS and Ikawa Y (1996) Genomic imprinting of the human serotonin-receptor (HTR2) gene involved in development of retinoblastoma. *Am J Hum Genet* **59**: 1084–1090

Kondo M, Suzuki H, Ueda R, Osada H, Takagi K and Takahashi T (1995) Frequent loss of imprinting of the H19 gene is often associated with its overexpression in human lung cancers. *Oncogene* **10**: 1193–1198

Labosky PA, Barlow DP and Hogan BLM (1994) Mouse embryonic germ (EG) cell lines: transmission through the germline and differences in the methylation imprint of insulin-like growth factor 2 receptor (Igf2r) gene compared with embryonic stem (ES) cell lines. *Development* **120**: 3197–3204

Leighton PA, Ingram RS, Eggenschwiler J, Efstratiadis A and Tilghman SM (1995) Disruption of imprinting caused by deletion of the H19 gene region in mice. *Nature* **375**: 34–39

Li E, Beard C and Jaenisch R (1993) Role of DNA methylation in genomic imprinting. *Nature* **366**: 362–365

Li XR, Adam G, Cui HM, Sandstedt B, Ohlsson R and Ekstroem TJ (1995) Expression, promoter usage and parental imprinting status of insulin-like growth factor II (IGF2) in human hepatoblastoma: uncoupling of IGF2 and H19 imprinting. *Oncogene* **11**: 221–229

Looijenga LHJ, Abraham M, Gillis AJM, Saunders GF and Oosterhuis JW (1994) Testicular germ cell tumors of adults show deletions of chromosomal bands 11p13 and 11p15.5, but no abnormalities within the zinc-finger regions and exons 2 and 6 of the Wilms' tumor 1 gene. *Genes Chromosome Cancer* **9**: 153–160

© Cancer Research Campaign 1997

Looijenga LHJ, Verkerk AJMH, De Groot N, Hochberg AA and Oosterhuis JW (1996) H19 in normal development and neoplasia. *Mol Reprod Devel* (in press)

Lothe RA, Hastie N, Heimdal K, Fosså SD, Stenwig AE and Børresen AL (1993) Frequent loss of 11p13 and 11p15 loci in male germ cell tumors. *Genes Chromosome Cancer* **7**: 96–101

Lothe RA, Peltomäki P, Tommerup N, Fossa SD, Stenwig AE, Borresen AL and Nesland JM (1995) Molecular genetic changes in human male germ cell tumors. *Lab Invest* **73**: 606–614

Luebbert M, Salser W, Prokocimer M, Miller CW, Thomason A and Koeffler HP (1991) Stable methylation patterns of MYC and other genes regulated during terminal myeloid differentiation. *Leukemia*, **5**: 533–539

Lustig O, Ariel I, Ilan J, Lev-Lehman E, De-Groot N and Hochberg A (1994) Expression of the imprinted gene H19 in the human fetus. *Mol Reprod Devel* **38**: 239–246

Maniatis T, Fritsch EF and Sambrook J (1982) Isolation of high molecular-weight, eukaryotic DNA from cells grown in tissue culture. In *Molecular Cloning*, p. 280. Cold Spring Harbor Laboratory: New York

McGrew MJ and Rosenthal N (1993) Quantitation of genomic methylation using ligation-mediated PCR. *BioTechniques* **15**: 722–729

Mishina M, Ogawa O, Kinoshita H, Oka H, Okumura K, Mitsumori K, Kakehi Y, Reeve AE and Yoshida O (1996) Equivalent parental distribution of frequently lost alleles and biallelic expression of the H19 gene in human testicular germ cell tumor. *Jpn J Cancer Res* **87**: 816–823

Monk M (1990) Changes in DNA methylation during mouse embryonic development in relation to X-chromosome activity and imprinting. *Phil Trans R Soc Lond* **326**: 299–312

Mostofi FK, Sesterhenn IA and Davis CJJ (1987) Immunopathology of germ cell tumors of the testis. *Sem Diagn Pathol* **4**: 320–341

Moulton T, Crenshaw T, Hao Y, Moosikasuwan J, Lin N, Dembitzer F, Hensle T, Weiss L, McMorrow L, Loew T, Kraus W, Gerald W and Tycko B (1994) Epigenetic lesions at the H19 locus in Wilms' tumour patients. *Nature Genet* **7**: 440–447

Mueller PR and Wold B (1989) In vivo footprinting of muscle specific enhancer by ligation mediated PCR. *Science* **246**: 780–786

Møller H, Jorgenson N and Forman D (1995) Trends in incidence of testicular cancer in boys and adolescent men. *Int J Cancer* **61**: 761–764

Oosterhuis JW and Looijenga LHJ (1993) The biology of human germ cell tumors: Retrospective speculations and new prospectives. *Eur Urol* **23**: 245–250

Peltomäki P (1991) DNA methylation changes in human testicular cancer. *Biochim Biophys Acta* **1096**: 187–196

Peng H-Q, Bailey D, Bronson D, Goss PE and Hogg D (1995) Loss of heterozygosity of tumor suppressor genes in testis cancer. *Cancer Res* **55**: 2871–2875

Pfeifer GP, Steigerwald SD, Mueller PR, Wold B and Riggs AD (1989) Genomic sequencing and methylation analysis by ligation mediated PCR. *Science* **246**: 810–813

Poirier F, Chan CTJ, Timmons PM, Robertson EJ, Evans MJ and Rigby PWJ (1991) The murine H19 gene is activated during embryonic stem cell differentiation in vitro and at the time of implantation in the developing embryo. *Development* **113**: 1105–1114

Rachmilewitz J, Gileadi O, Eldar-Geva T, Schneider T, de Groot N and Hochberg A (1992) Transcription of the H19 gene in differentiating cytotrophoblasts from human placenta. *Molec Reprod Devel* **32**: 196–202

Rachmilewitz J, Elkin M, Looijenga LHJ, Verkerk AJMH, Gonik B, Lustig G, Werner D, de Groot N and Hochberg A (1996) Characterization of the imprinted IPW gene: allelic expression in normal and tumorigenic human tissues. *Oncogene* **13**: 1687–1692

Rainier S, Johnson LA, Dobry CJ, Ping AJ, Grundy PE and Feinberg AP (1993) Relaxation of imprinted genes in human cancer. *Nature* **362**: 747–749

Rainier S, Dobry CJ and Feinberg AP (1995) Loss of imprinting in hepatoblastoma. *Cancer Res* **55**: 1836–1838

Razin A and Shemer R (1995) DNA methylation in early development. *Hum Mol Genet* **4**: 1751–1755

Reik W and Allen ND (1994) Genomic imprinting: imprinting with and without methylation. *Curr Biology* **4**: 145–147

Reik W, Brown KW, Slatter RE, Sartori P, Elliott M and Maher ER (1994) Allelic methylation of H19 and IGF2 in the Beckwith–Wiedemann syndrome. *Hum Mol Genet* **3**: 1297–1301

Riou G, Ahomadegbe JC, Tourpin S and Douc-Rasy S (1996) Loss of imprinting of H19 and IGF2 genes is associated with inflammatory breast carcinoma (Abstract). *Ann Oncol* **7**: 4

Sasaki H, Ferguson-Smith AC, Shum ASW, Barton SC and Surani MA (1995) Temporal and spatial regulation of H19 imprinting and uniparental mouse embryos. *Development* **121**: 4195–4202

Shemer R, Kafri T, O'Connell A, Eisenberg S, Breslow JL and Razin A (1991) Methylation changes in the apolipoprotein AI gene during embryonic development of the mouse. *Proc Natl Acad Sci USA* **88**: 11300–11304

Skakkebæk NE, Berthelsen JG, Giwercman A and Müller J (1987) Carcinoma-in-situ of the testis: possible origin from gonocytes and precursor of all types of germ cell tumours except spermatocytoma. *Int J Androl* **10**: 19–28

Steenman MJC, Rainier S, Dobry CJ, Grundy P, Horon IL and Feinberg AP (1994) Loss of imprinting of IGF2 is linked to reduced expression and abnormal methylation of H19 in Wilms' tumour. *Nature Genet* **7**: 433–439

Stöger R, Kubicka P, Liu CG, Kafri T, Razin A, Cedar H and Barlow DP (1993) Maternal-specific methylation of the imprinted mouse igf2r locus identifies the expressed locus as carrying the imprinting signal. *Cell* **73**: 61–71

Svensson K, Walsh C, Fundele R and Ohlsson R (1995) H19 is imprinted in the choroid plexus and leptomeninges of the mouse foetus. *Mech Devel* **51**: 31–37

Szabo PE and Mann JR (1994) Expression and methylation of imprinted genes during in vitro differentiation of mouse parthenogenetic and androgenetic embryonic stem cell lines. *Development* **120**: 1651–1660

Szabo PE and Mann JR (1995) Biallelic expression of imprinted genes in the mouse germ line: implications for erasure, establishment, and mechanisms of genomic imprinting. *Genes Devel* **9**: 1857–1868

Taniguchi T, Sullivan MJ, Ogawa O and Reeve AE (1995) Epigenetic changes encompassing the IGF2/H19 locus associated with relaxation of IGF2 imprinting and silencing of H19 in Wilms tumor. *Proc Natl Acad Sci USA* **92**: 2159–2163

Tremblay KD, Saam JR, Ingram RS, Tilghman SM and Bartolomei MS (1995) A paternal-specific methylation imprint marks the alleles of the mouse H19 gene. *Nature Genet* **9**: 407–413

Uyeno S, Aoki Y, Nata M, Sagisaka K, Kayama T, Yoshimoto T and Ono T (1996) IGF2 but not H19 shows loss of imprinting in human glioma. *Cancer Res* **56**: 5356–5359

Van Gurp RJLM, Oosterhuis JW, Kalscheuer V, Mariman ECM and Looijenga LHJ (1994) Human testicular germ cell tumors show biallelic expression of the H19 and IGF2 gene. *J Natl Cancer Inst* **86**: 1070–1075

Verkerk AJMH, Ariel I, Dekker MC, Schneider T, van Gurp Rjlm, DE Groot N, Gillis AJM, Oosterhuis JW, Hochberg AA and Looijenga LHJ (1996) Unique expression patterns of H19 in human testicular cancers of different etiology. *Oncogene* **14**: 95–107

Walsh C, Miller SJ, Flam F, Fisher RA and Ohlsson R (1995) Paternally derived H19 is differentially expressed in malignant and nonmalignant trophoblast. *Cancer Res* **55**: 1111–1116

Zhan S, Shapiro DN and Helman LJ (1994) Activation of an imprinted allele of the insulin-like growth factor II gene implicated in rhabdomyosarcoma. *J Clin Invest* **94**: 445–448

Zhan SL, Shapiro DN and Helman LJ (1995) Loss of imprinting of IGF2 in Ewing's sarcoma. *Oncogene* **11**: 2503–2507

Zhang Y and Tycko B (1992) Monoallelic expression of the human H19 gene. *Nature Genet* **1**: 40–44

Zhang Y, Shields T, Crenshaw T, Hao Y, Moulton T and Tycko B (1993) Imprinting of human H19: allele-specific CpG methylation, loss of the active allele in Wilms tumor, and potential for somatic allele switching. *Am J Hum Genet* **53**: 113–124

© Cancer Research Campaign 1997